# Intensive Care Unit Liberation After Complete Left Anterior Descending Artery Occlusion: Unexpected Neurologic Recovery After a 53-Minute Cardiac Arrest

**DOI:** 10.7759/cureus.91751

**Published:** 2025-09-06

**Authors:** Sabrina M Miller, Zechariah Jean, Jennifer A Kyko, Imad Obeid, Michael Buggia

**Affiliations:** 1 Emergency Medicine, Michigan State University College of Osteopathic Medicine, Warren, USA; 2 Emergency Medicine, Henry Ford Health System, Warren, USA; 3 Intensive Care Unit, Henry Ford Health System, Warren, USA

**Keywords:** anoxic brain injury, complete left anterior descending artery occlusion, neurologic recovery, out-of-hospital cardiac arrest, prolonged return of spontaneous circulation

## Abstract

Out-of-hospital cardiac arrest (OHCA) is associated with low survival and neurologic recovery rates, especially when return of spontaneous circulation exceeds 45 minutes. Additionally, neurologic prognostic indicators, such as absent brainstem reflexes and presence of alpha coma electroencephalogram (EEG) patterns, are typically associated with poor outcomes. This report outlines the case of a 48-year-old woman with a history of hypertension, dyslipidemia, and tobacco use who suffered an OHCA due to an acute left anterior descending artery occlusion. She experienced a total downtime of 53 minutes and required multiple rounds of defibrillation and advanced cardiac life support. Following successful percutaneous coronary intervention, her initial neurologic examination remained poor with absent brainstem reflexes, nonreactive pupils, and EEG findings consistent with severe encephalopathy. After 22 days in the hospital with minimal neurologic improvement, she was discharged to long-term care with a tracheostomy and percutaneous endoscopic gastrostomy tube. Two months after initial discharge, the patient unexpectedly returned to the hospital, demonstrating signs of cognitive recovery. During this second hospital stay, she was alert, followed commands, was able to communicate using a voice modulator, and passed a swallow study. This case illustrates a rare example of survival after prolonged OHCA and subsequent neurologic improvement following an initially guarded prognosis.

## Introduction

Out-of-hospital cardiac arrest (OHCA) remains a major public health concern with persistently low survival and neurologic recovery rates. The American Heart Association reports that survival rates from hospital to discharge after suffering an OHCA range from 6% to 10% [1]. Among those who survive, favorable neurologic outcomes are achieved in only 8% of cases [2]. A favorable neurologic outcome is commonly defined by Cerebral Performance Category (CPC) scores of 1 or 2, with scores of 3 and 4 indicative of poor neurologic function [3]. Neurologic injury is a major cause of morbidity and mortality following cardiac arrest, often resulting from global cerebral hypoxia during prolonged downtime and incomplete resuscitation [2].

Time to return of spontaneous circulation (ROSC) is one of the most important prognostic factors, with prolonged cardiopulmonary resuscitation (CPR) durations reducing the chance of survival and meaningful neurologic recovery [1,4]. Studies show that the likelihood of favorable neurologic recovery decreases after 30-45 minutes of CPR, and after 50 minutes of downtime, fewer than 1% of patients achieve CPC scores of 1 or 2 [3,4]. Additionally, most survivors who regain meaningful neurologic function do so within the first six months after cardiac arrest [5,6]. Patients with initially poor neurologic examinations, such as absent brainstem reflexes and electroencephalograms (EEGs) demonstrating alpha coma patterns, are typically considered to have a poor prognosis with minimal chance of improvement [2,7].

This report describes the case of a 48-year-old woman who suffered an OHCA due to an acute left anterior descending (LAD) artery occlusion, experienced a total downtime of 53 minutes, and initially exhibited findings consistent with severe anoxic brain injury. Despite these early poor prognostic factors, she later demonstrated delayed but meaningful neurologic recovery. This case highlights the potential for delayed neurologic improvement, particularly in younger patients with a strong prior functional status.

## Case presentation

A 48-year-old African American female with a past medical history of hypertension, dyslipidemia, tobacco use, and a family history of premature coronary artery disease presented to the emergency department (ED) in cardiac arrest. Of note, the patient had undergone an exercise stress echocardiogram two months before her presentation that showed a preserved left ventricular ejection fraction of 60% with no inducible ischemia or wall motion abnormalities.

While shopping at a retail store, bystanders reported seizure-like activity followed by the patient collapsing. Bystanders at the retail store immediately initiated CPR and called Emergency Medical Services (EMS). Upon arrival, EMS found the patient in ventricular fibrillation (VF) and initiated advanced cardiac life support (ACLS). The patient was intubated in the field, and EMS placed a LUCAS chest compression device to provide mechanical compressions during transport. The estimated prehospital downtime was approximately 30 minutes.

Upon arrival at the ED, the patient remained in VF without a pulse. An additional 15 minutes of ACLS was performed, including delivery of five defibrillation shocks. Medications administered included amiodarone, epinephrine, magnesium, bicarbonate, and calcium were administered. ROSC was achieved after 45 minutes of ACLS.

**Figure 1:**
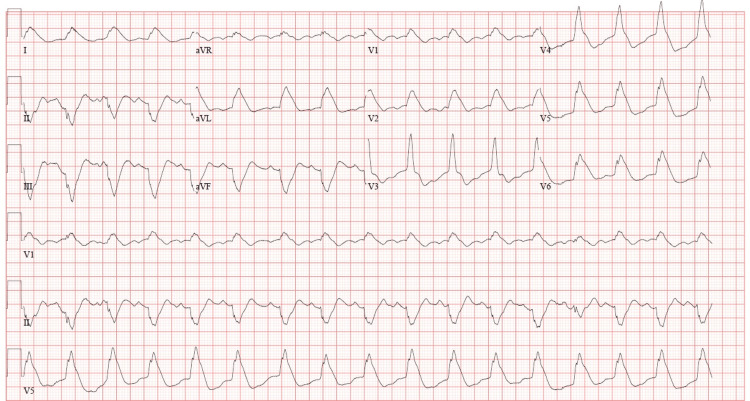
Initial 12-lead electrocardiogram obtained in the emergency department demonstrating diffuse ST-segment elevations consistent with an acute ST-elevation myocardial infarction.

**Figure 2:**
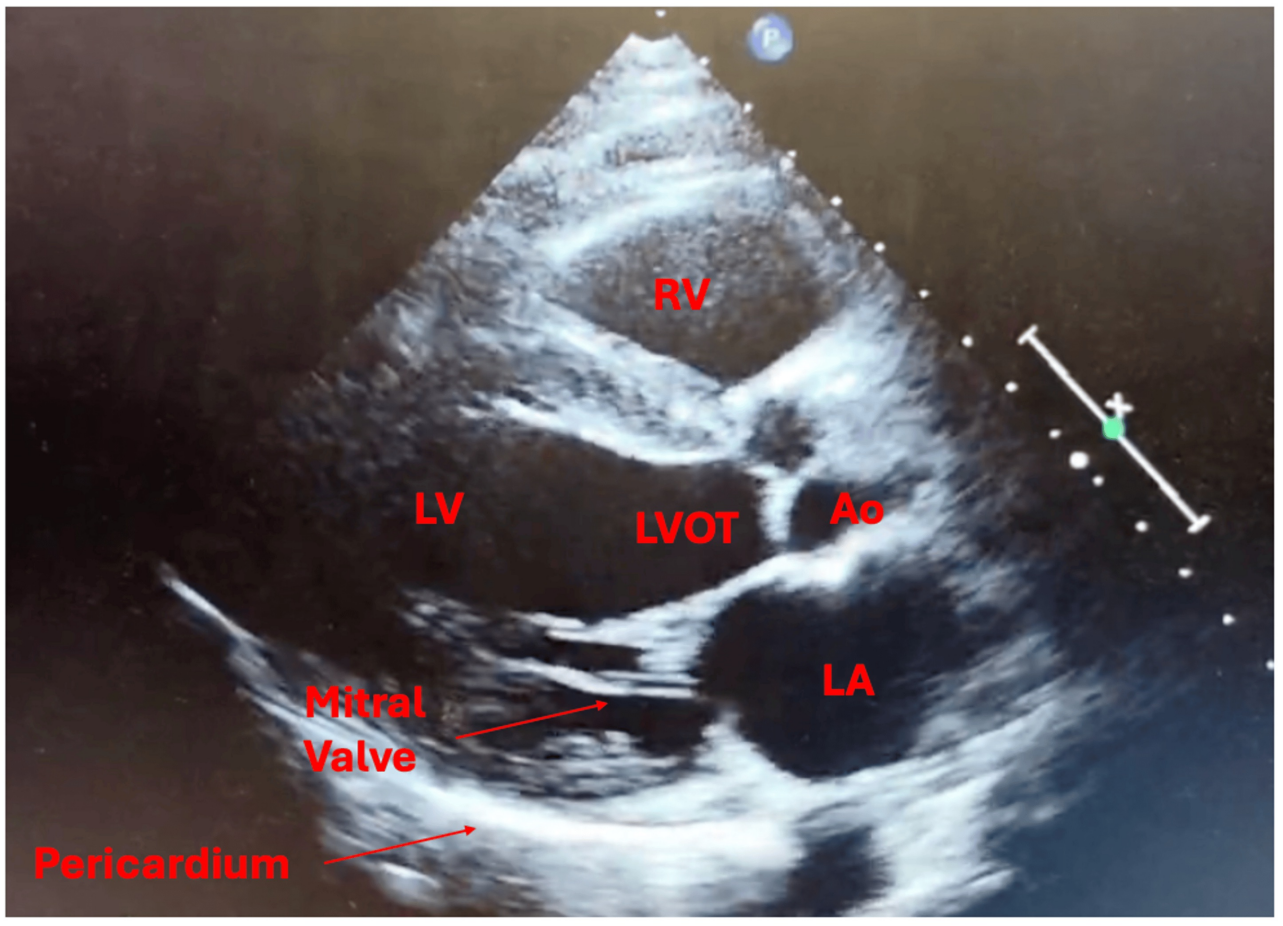
Parasternal long-axis transthoracic echocardiogram. The RV is not dilated, consistent with no RV strain. The pericardium is visualized without evidence of pericardial effusion. RV: right ventricle; LV: left ventricle; LA: left atrial; LVOT: left ventricular outflow tract

An initial ECG showed a wide complex tachycardia with diffuse ST elevations, consistent with ST-elevation myocardial infarction (Figure 1). A bedside echocardiogram obtained following initial ROSC demonstrated a low ejection fraction of approximately 20%, without signs of pericardial effusion or right ventricular strain (Figure 2). Approximately three minutes later, the patient returned to VF. The patient received one additional defibrillation shock and underwent eight more minutes of CPR with continued ACLS before the patient reestablished ROSC. This brought the patient’s total estimated downtime to 53 minutes. Cardiology was urgently consulted, and the patient was transferred for emergent cardiac catheterization.

**Figure 3:**
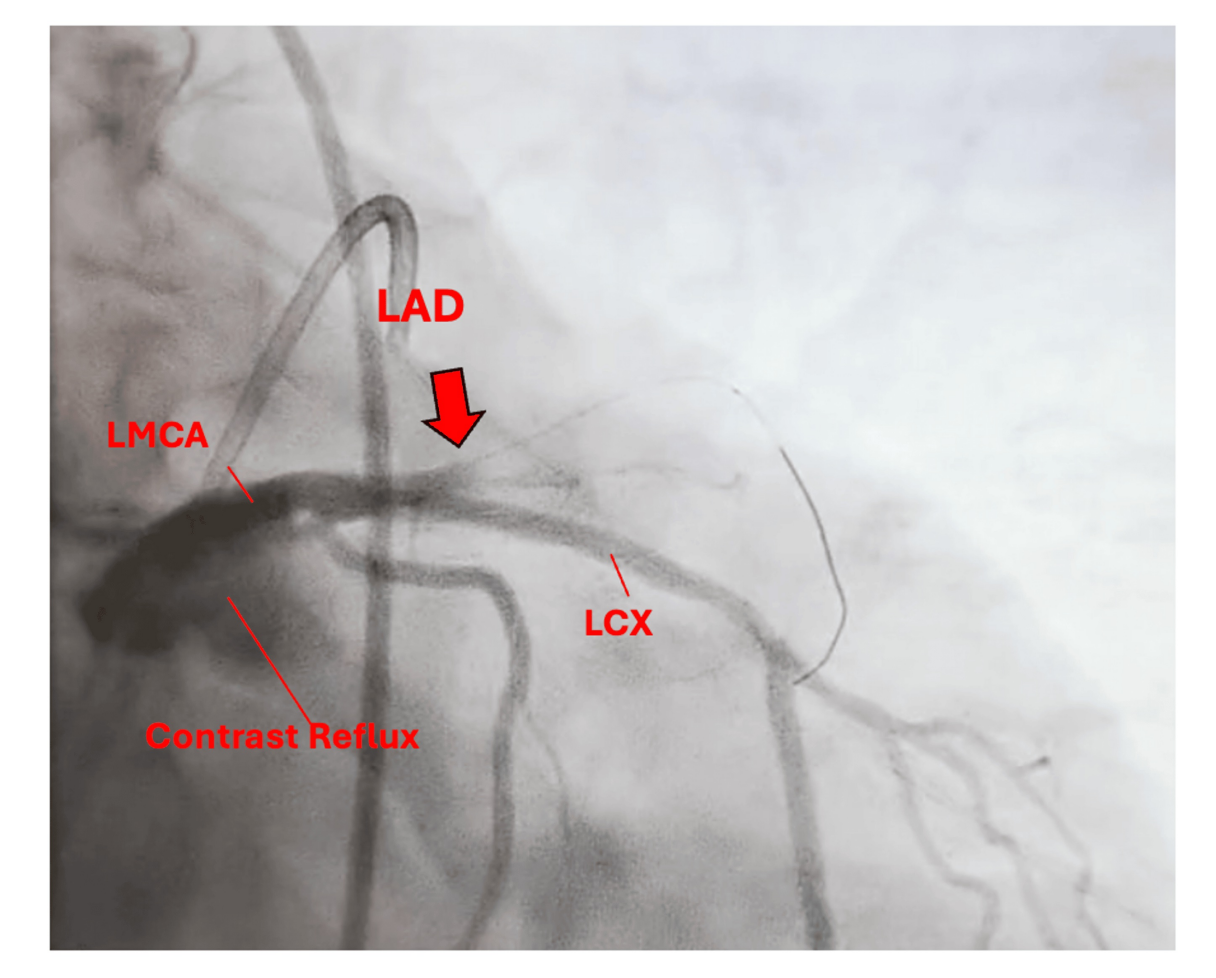
Coronary angiography showing a 100% acute occlusion of the proximal to mid-LAD artery (red arrow). Contrast reflux into the LMCA is visible, consistent with complete obstruction. LAD: left anterior descending; LMCA: left main coronary artery; LCX: left circumflex

**Figure 4:**
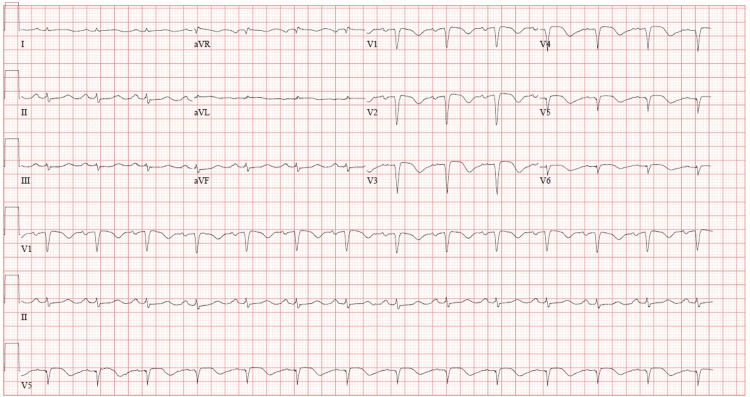
Post-percutaneous coronary intervention ECG demonstrating resolution of diffuse ST-segment elevations following left anterior descending artery revascularization.

Coronary angiography revealed a 100% acute occlusion of the proximal to mid-left LAD artery (Figure 3). Percutaneous coronary intervention (PCI) with stent placement was successfully performed. The procedure was complicated by five additional episodes of ventricular tachycardia and VF, requiring defibrillation. The patient’s left ventricular end-diastolic pressure was elevated at 30 mmHg, and the patient’s arrhythmia frequency decreased following successful revascularization of the LAD (Figure 4).

The patient was subsequently admitted to the intensive care unit (ICU) for post-cardiac arrest care. On initial neurologic examination, the patient was intubated and not sedated. The patient’s pupils were 3 mm bilaterally and nonreactive to light, her corneal reflexes were absent, her oculocephalic reflex was weakly present, and her gag reflex was intact. The patient exhibited occasional spontaneous respirations over the ventilator. The patient’s deep tendon and plantar reflexes were absent, her muscle tone was decreased, and she demonstrated intermittent generalized myoclonic jerking. Continuous EEG monitoring was ordered to evaluate for myoclonic status epilepticus, and antiepileptic therapy was initiated with intravenous midazolam and levetiracetam.

Initial laboratory evaluation revealed a metabolic acidosis (pH of 7.19), elevated lactic acid (11.2 mmol/L), hyponatremia (125 mmol/L), and elevated troponin (194 ng/L). A non-contrast head CT revealed no acute intracranial hemorrhage or mass effect.

**Figure 5:**
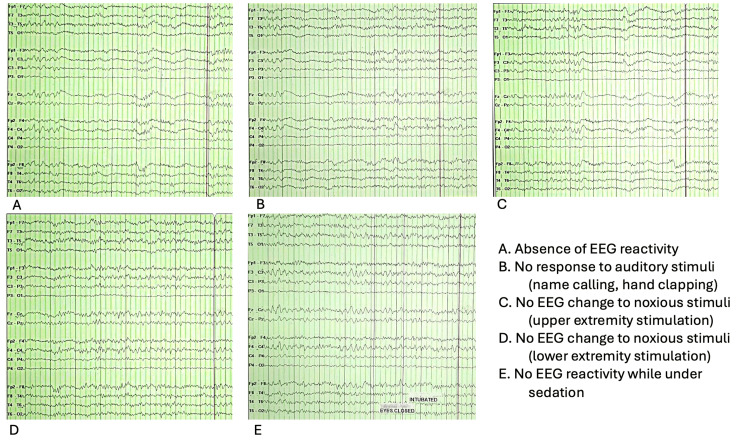
Absence of EEG reactivity to multiple stimuli. EEG tracings from a comatose patient demonstrating no change in background activity following (A) baseline observation, (B) auditory stimulus (technician calling name and hand clapping), (C) noxious stimulus to bilateral upper extremities, (D) noxious stimulus to lower extremities, and (E) sedation. EEG: electroencephalogram

The patient remained in the ICU for 17 days and showed minimal neurologic improvement, unable to follow commands, lacking purposeful movements, and demonstrating only intermittent opening of her eyes. Serial EEGs performed on hospital days 2, 8, 12, and 16 demonstrated severe encephalopathy with nonreactive patterns and alpha coma patterns (Figure 5). Although the patient passed a spontaneous breathing trial on hospital day seven, extubation was deferred due to persistently low Glasgow Coma Scale scores. A tracheostomy was performed on hospital day 13 for ventilator dependence, and a percutaneous endoscopic gastrostomy (PEG) tube was placed on hospital day 17 for nutritional support. She was discharged on hospital day 22 to a long-term acute care facility with the tracheostomy and PEG tube in place. The patient exhibited minimal neurologic function upon discharge to the long-term acute care facility.

Two months after initial discharge, the patient returned to the ED after falling from her bed. On initial examination in the ED, the patient demonstrated improved neurologic findings since her previous hospital discharge two months prior. The patient was alert and able to localize to pain, although her verbal responses were inappropriate. By hospital day three, the patient was more alert, responding to verbal stimuli, and communicating using a tracheostomy collar with a voice modulator.

By hospital day eight, the patient continued to improve, demonstrating orientation to self, the ability to follow simple commands, feed herself thick liquids, identify her sister, and name her pet. The patient was discharged in stable condition on hospital day eight.

Seven months after the initial hospitalization, the patient continues to demonstrate meaningful neurologic recovery. She successfully passed a swallow study and was cleared for an oral diet.

## Discussion

Among those who survive an OHCA, neurologic recovery is highly variable. Studies estimate that only 12% of patients survive to hospital discharge, and approximately 8% achieve favorable neurologic outcomes, as defined by CPC scores of 1-2 [2].

Time to ROSC is a critical predictor of both survival and neurologic recovery. Studies have demonstrated that shorter time to ROSC, particularly when achieved on scene, is associated with significantly improved outcomes [4]. One study found that patients with favorable neurologic outcomes were younger (mean age: 55.9 ± 14.7) and had significantly shorter CPR durations compared to those with unfavorable neurologic outcomes (mean age: 62.1 ± 12.7) [4]. When ROSC occurred between 30-44 minutes, only 15% of patients had favorable neurologic outcomes; beyond 45 minutes, only 18% of patients demonstrated favorable neurologic outcomes [4].

A large retrospective cohort study evaluating approximately 350,000 in-hospital cardiac arrest similarly demonstrated a sharp decline in survival and neurologic recovery with increasing CPR duration [3]. The probability of a favorable neurological outcome (CPC scores of 1-2) dropped to less than 1% after 32 minutes of CPR, and a survival to discharge fell below 1% after 39 minutes. Among patients who received more than 50 minutes of CPR, survival to discharge was just 0.5%, with favorable neurologic outcomes occurring in fewer than 0.4% of patients under 60 years old [3].

Delayed neurologic improvement after cardiac arrest is particularly rare in patients discharged with poor neurological function. In one study, only two out of the 48 survivors with an unfavorable neurologic outcome (CPC score of 3) one month after cardiac arrest improved to a favorable neurologic outcome (CPC score of 2) within six months of follow-up [6]. Additionally, in a separate study, nine (3.6%) patients improved to CPC scores of 1 or 2 within six months after cardiac arrest, but neurologic improvements were not seen past a six-month period [5].

The case presented here represents an example of both unlikely survival and delayed neurologic recovery after prolonged cardiac arrest. Despite a total estimated downtime of 53 minutes, nonreactive pupils, and serial EEGs showing severe and nonreactive encephalopathy with alpha coma patterns, the patient ultimately regained meaningful cognitive and functional abilities, including speech, orientation, verbalization, and the ability to feed herself soft foods. A brief review of the literature suggests that certain factors, such as her relatively young age and strong prior functional status, may have contributed to this outcome. This case highlights the potential for neurologic improvement beyond the acute phase and challenges the initial neurologic prognosis traditionally associated with anoxic brain injury following prolonged cardiac arrest.

## Conclusions

This case demonstrates the unpredictable nature of neurologic recovery following prolonged OHCA, challenging initial prognostic expectations. It illustrates that the distinction between favorable and unfavorable prognostic indicators is not absolute and may vary based on individual patient factors and family perspectives. Ultimately, prognostication should be approached with humility, recognizing that individual patient outcomes may defy established medical expectations.

## References

[1] Tamis-Holland JE, Menon V, Johnson NJ (2024). Cardiac catheterization laboratory management of the comatose adult patient with an out-of-hospital cardiac arrest: a scientific statement from the American Heart Association. Circulation.

[2] Geocadin RG, Callaway CW, Fink EL (2019). Standards for studies of neurological prognostication in comatose survivors of cardiac arrest: a scientific statement from the American Heart Association. Circulation.

[3] Okubo M, Komukai S, Andersen LW, Berg RA, Kurz MC, Morrison LJ, Callaway CW (2024). Duration of cardiopulmonary resuscitation and outcomes for adults with in-hospital cardiac arrest: retrospective cohort study. BMJ.

[4] Braumann S, Nettersheim FS, Hohmann C (2020). How long is long enough? Good neurologic outcome in out-of-hospital cardiac arrest survivors despite prolonged resuscitation: a retrospective cohort study. Clin Res Cardiol.

[5] Hayamizu M, Kodate A, Sageshima H (2023). Delayed neurologic improvement and long-term survival of patients with poor neurologic status after out-of-hospital cardiac arrest: a retrospective cohort study in Japan. Resuscitation.

[6] Kim YJ, Ahn S, Sohn CH (2016). Long-term neurological outcomes in patients after out-of-hospital cardiac arrest. Resuscitation.

[7] Fischer D, Edlow BL (2024). Coma prognostication after acute brain injury: a review. JAMA Neurol.

